# InN Based Water Condensation Sensors on Glass and Flexible Plastic Substrates

**DOI:** 10.3390/s131216940

**Published:** 2013-12-06

**Authors:** Viorel Dumitru, Stefan Costea, Mihai Brezeanu, George E. Stan, Cristina Besleaga, Aurelian C. Galca, Gabriela Ionescu, Octavian Ionescu

**Affiliations:** 1 Honeywell Romania S.R.L, Sensors & Wireless Laboratory Bucharest, Calea Floreasca 169A, Building A, Bucharest 014459, Romania; E-Mails: stefan.costea@honeywell.com (S.C.); mihai.brezeanu@honeywell.com (M.B.); 2 National Institute of Materials Physics, Atomistilor 105 bis, Magurele 077125, Romania; E-Mails: george_stan@infim.ro (G.E.S.); cristina.besleaga@infim.ro (C.B.); ac_galca@infim.ro (A.C.G.); 3 Petroleum-Gas University of Ploiesti, Blvd. Bucuresti no 39, Ploieşti 100680, Romania; E-Mails: ionescug@upg-ploiesti.ro (G.I.); ionescu_o_ro@yahoo.com (O.I.)

**Keywords:** III-nitride, indium nitride, condensation sensor, surface accumulation layer

## Abstract

In this paper, we report the realization and characterization of a condensation sensor based on indium nitride (InN) layers deposited by magnetron sputtering on glass and flexible plastic substrates, having fast response and using potentially low cost fabrication technology. The InN devices work as open gate thin film sensitive transistors. Condensed water droplets, formed on the open gate region of the sensors, deplete the electron accumulation layer on the surface of InN film, thus decreasing the current of the sensor. The current increases back to its initial value when water droplets evaporate from the exposed InN film surface. The response time is as low as 2 s.

## 1. Introduction

In the recent years, indium nitride (InN) has attracted increased attention and research efforts due to its reported properties that make it suitable for a wide range of applications [1–5]. InN has also shown significant potential for sensing applications: room temperature hydrogen detection [6,7], anion detection [8], calcium ions sensing [9], pH measurement [10], and sub-ppm acetone detection [11] were reported.

In this paper, we report the realization and characterization of a fast and potentially low cost condensation sensor technology based on InN layers deposited by magnetron sputtering on glass and flexible plastic substrates. Condensation sensors are required in many applications, including condensation monitoring in Heating, Ventilation and Air Conditioning (HVAC) installations, prevention of “indoor rain” caused by chilled surfaces, and detection of water condensation on windshields. Water detection using III-nitride layers is well established [12]: the typical sensing structure is based on gate-less AlGaN/GaN high electron mobility transistor (HEMT) structures epitaxially grown by metal organic chemical vapor deposition (MOCVD) on sapphire substrates and processed using standard photolithographic technology. However, the fabrication of this sensing structure is expensive, limiting the mass production of these sensors.

## 2. Experimental Section

In our study, InN films were deposited on different substrates: glass, polyethylene terephthalate (PET) and silicon (111). The deposition was performed by reactive radio-frequency magnetron sputtering from a high purity indium target (99.9999%, Mateck GmbH, Juelich, Germany), with a thickness of 3 and 110 mm in diameter. Before being introduced into the deposition chamber, the substrates were ultrasonically cleaned in isopropyl alcohol for 10 min, and then dried in an argon flow. The target-to-substrate separation distance was set to 35 mm. The deposition chamber was initially evacuated down to a base pressure of ∼2 × 10^−4^ Pa, and then two high purity working gases—Ar and N_2_—were admitted. The total gas flow was 40 sccm, at a selected working pressure of 0.95 Pa, with a nitrogen partial pressure of 65%. Prior to the deposition, the target was sputter-cleaned for 20 min in argon (0.3 Pa) and then another 10 min in the working atmosphere (argon-nitrogen mix), in order to stabilize the reactive sputtering process and activate the target. Further on, the substrates were processed by etching (using a protocol described elsewhere [13]), in order to remove the native oxide and other impurities that might have still been present after the ultrasonic cleaning, and to increase the film adherence.

To avoid overheating, a low power (∼100 W) setting was used on the RF generator (1.78 MHz). Although no external heating was used during the InN deposition, the substrate temperature reached 50 °C at the end of the deposition cycle due to plasma self-heating. After a deposition process that lasted 40 min, a 1.8 μm thick InN layer was obtained on the three different aforementioned substrates.

The crystalline structure and the morphology of the deposited InN layers were investigated by X-ray Diffraction (XRD) and Scanning Electron Microscopy (SEM).

**Figure 1.**
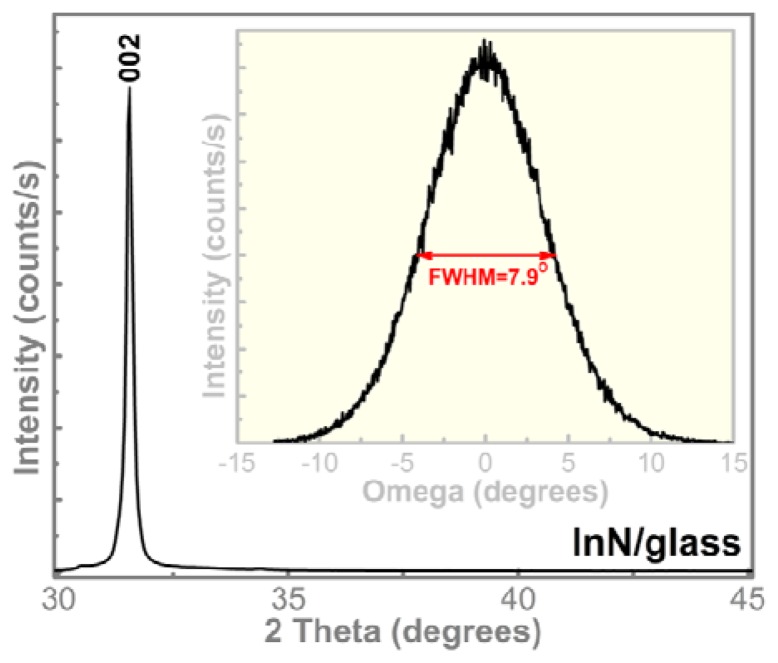
XRD pattern of the InN film deposited on glass. The inset shows (002) XRD rocking curve.

**Figure 2.**
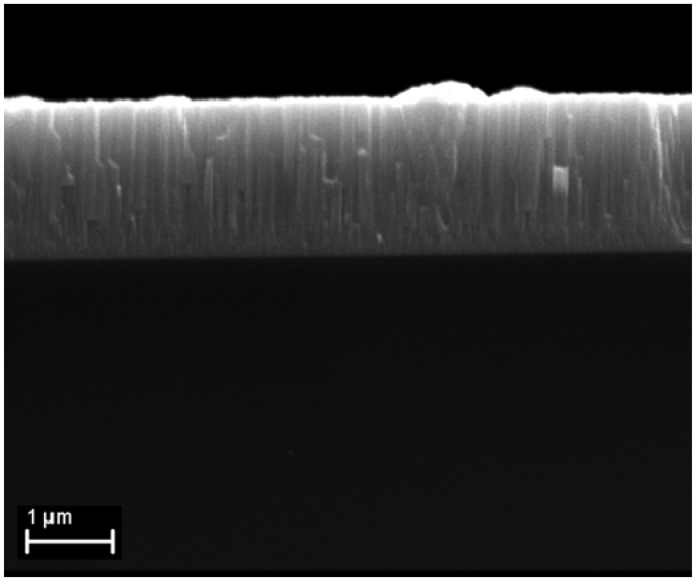
SEM image of the InN layer sputtered on Si (111).

The XRD results in Figure 1 indicate that the obtained layers have highly oriented InN polycrystalline structure (ICDD: 70-2547) with the *c*-axis perpendicular to the substrate. As could be seen, only the peak at ∼31° could be observed, that is the peak from (002) InN [14–17]. It should be highlighted that similar highly oriented structures were obtained on all three substrates [silicon (111), glass and PET], with full width at half maximum (FWHM) of (002) XRD rocking curve values around 8°. This is in good agreement with the cross-section SEM micrograph (Figure 2) which indicates that the layers have a compact structure, with a well-defined columnar morphology.

Further on, the sensor fabrication was finalized by evaporating Al electrodes using shadow masks on the InN layer previously deposited on glass and PET. The connecting wires were then bonded with silver paste on the Al contacts. In a similar way, we prepared samples for the characterization of the deposited InN layers by means of resistivity and Hall measurements. The results indicate that the deposited InN layers have a high electron concentration (*n* = 6.2 × 10^20^ cm^−3^), an electron mobility of 8 cm^2^/V·s, and a resistivity of 1.27 × 10^−3^ Ohm cm. The low electron mobility value obtained indicates poor transport properties of the polycristalline columnar films, with a lot of grain boundaries and defects. The columnar structure may result in an effective conductivity that is much lower that expected due to the high specific area. Unlike for the epitaxialy grown, high crystaline quality InN thin films, electron mobility values reported for the sputtered polycrystalline InN films, are low [18,19].

**Figure 3.**
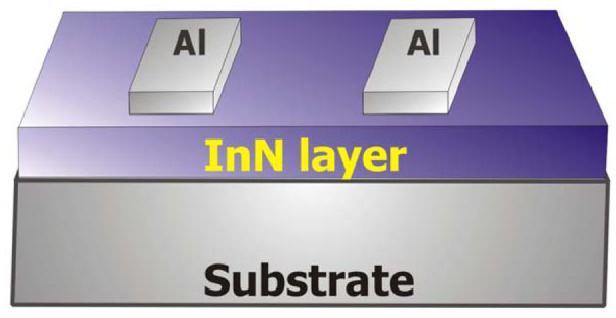
Schematic diagram of the fabricated InN-on-PET condensation sensor. The contact pads (Al) have an area of 2 mm^2^× 1 mm^2^ and the distance between them is 2 mm.

A schematic representation of the sensors realized on PET is shown in Figure 3. The sensor realized on glass was similar but with a different contact geometry. The sensors were operated as open gate thin film transistors with the gate region, between the source and drain Al contacts, left open to the environment.

## 3. Results and Discussion

The water condensation detection was tested by applying a constant bias voltage (0.01 V) between the two Al pads and monitoring the current flowing through the sensor, when water condensation occurs on the open gate region of the sensor. For this test, the InN sensors together with standard (commercial) humidity and temperature sensors were placed in a sealed box with a transparent cover. A cup of warm water (at 30–40 °C) was also placed inside the box. The humidity started to increase inside the box and, after a while, water condensation droplets occurred both at the surface of the InN sensor and on the box walls. Then, the box was opened and the droplets evaporated. The procedure was then repeated.

**Figure 4.**
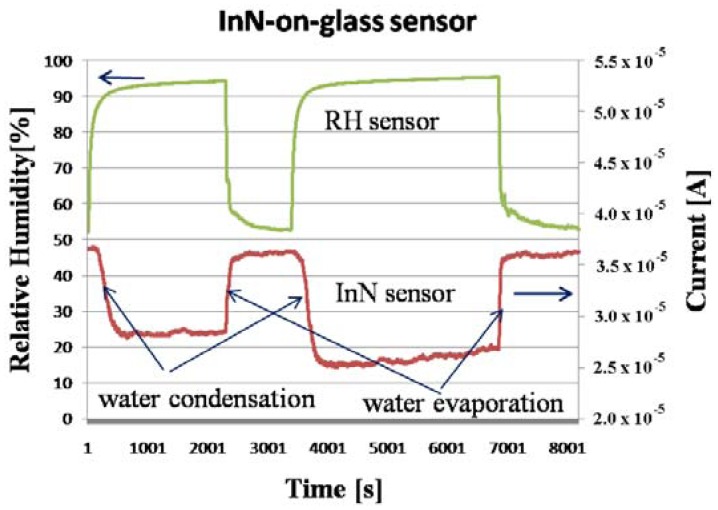
Current behavior during water condensation and evaporation for the InN-on-glass sensor.

**Figure 5.**
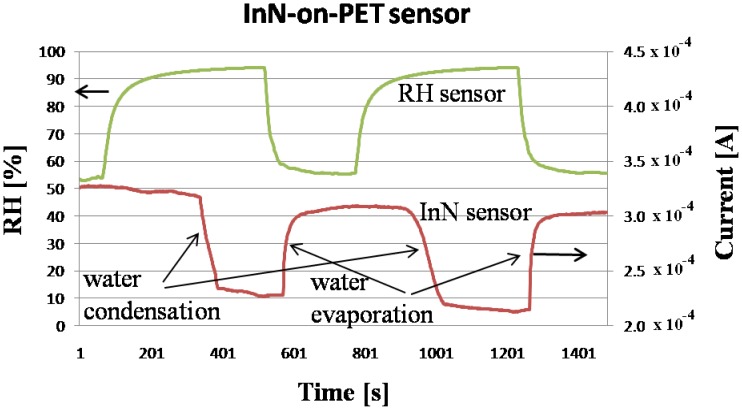
Current behavior during water condensation and evaporation for the InN-on-PET sensor.

**Figure 6.**
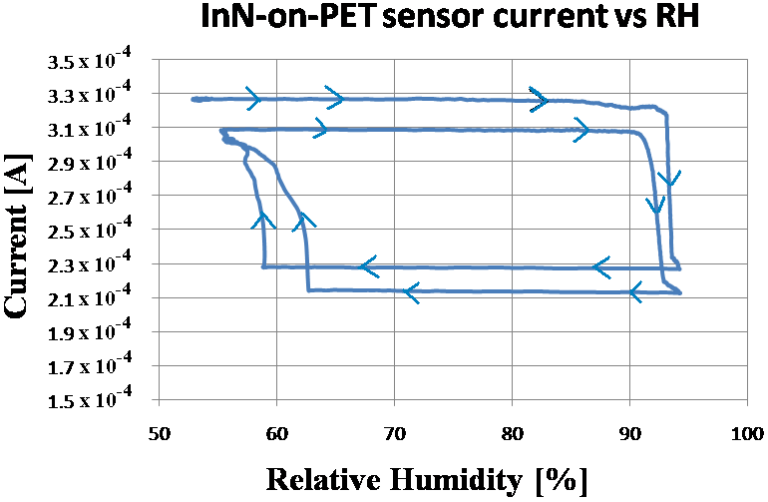
Sensor signal (current) *versus* relative humidity for the InN-on-PET sensor. The arrows indicate the succession in time measured during the experiment.

During the experiment, the monitored temperature inside the box varied between a 23.5 and 26.5 °C. The evolution of the measured current, flowing through the InN-based sensors during the experiment, is displayed in Figures 4, 5 and 6, together with the relative humidity (RH) signal from the standard reference sensor. The water condensation process occurring on the sensor open gate region leads to the decrease of the current flowing between the two Al contacts. As the water droplets evaporate, the current increases. The phenomenon is reversible and the current gets back to its initial value for the InN sensor built on glass (Figure 4) and close to that value for the sensor fabricated on PET (Figure 5). This difference might be due to the incomplete water evaporation in the latter case, due to the porosity of this film. The water is trapped in the pores and does not have time to evaporate. Therefore, in the second cycle, the water droplets condensing on the film surface are added to the ones still present in pores, resulting in larger area covered in water and a lower value for the current. Further investigations are needed in order to fully clarify the observed drift for the InN-on-PET sensor (Figure 5). At the same time, the sensor signal *versus* relative humidity plot inserted in Figure 6 shows typical behavior for a condensation sensor, with a cyclical switching of the sensor signal (current) when water is condensing and then re-evaporating on its surface. It can be seen that our samples switch off (current decreases) at about 90% RH, and switch back on when the relative humidity decreases below 60%. The noise observed on the switching thresholds is below 4% RH, much less than the difference between the thresholds. The detection is reliable despite the observed sensor drift.

**Figure 7.**
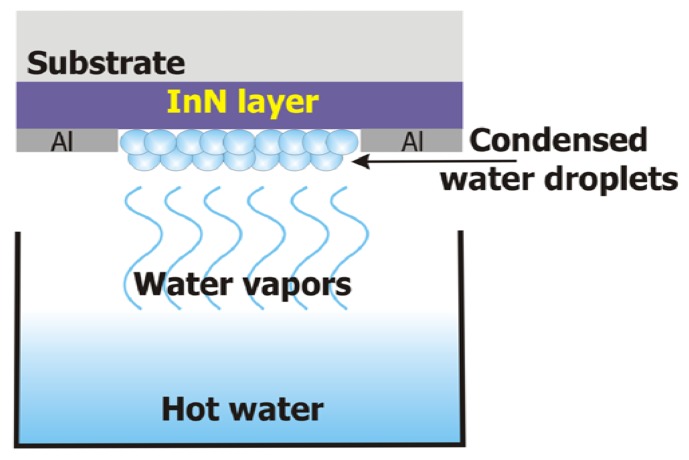
Schematic diagram of the set-up used for testing the response time of the InN-based condensation sensors.

Further on, in order to test how fast the sensors can respond to water condensation for applications where rapid water condensation detection is required, an InN-on-glass and an InN-on-PET sensor were placed faced up-side down above a cup with hot water (70 °C), as schematically depicted in Figure 7.

**Figure 8.**
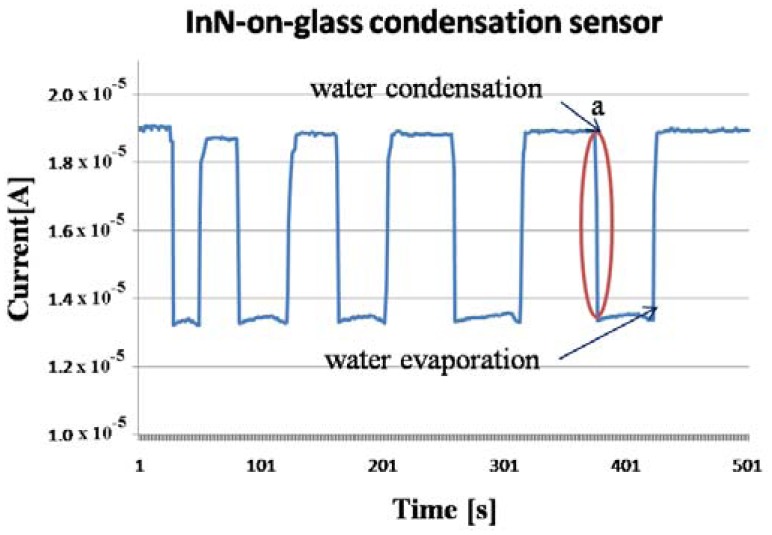
InN-on-glass condensation sensor signal at rapid water condensation and evaporation on its surface. Region a is detailed in Figure 9.

**Figure 9.**
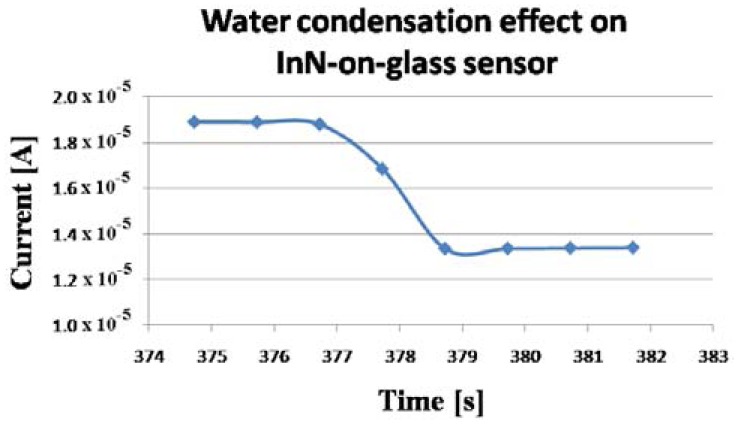
Current decrease during water condensation for the InN-on-glass condensation sensor. This corresponds to region a in Figure 8.

**Figure 10.**
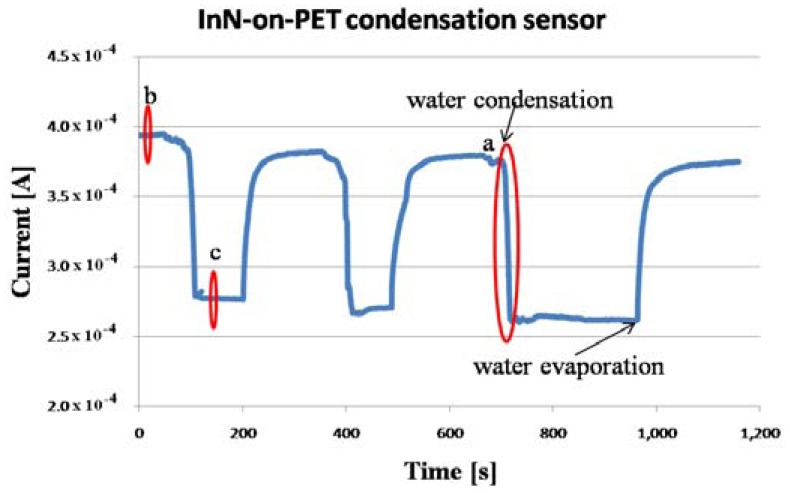
InN-on-PET condensation sensor signal at rapid water condensation and evaporation on its surface. Region a is detailed in Figure 11. In regions b and c, the measurement was interrupted for few seconds and the I–V characteristics displayed in Figure 12 were recorded.

**Figure 11.**
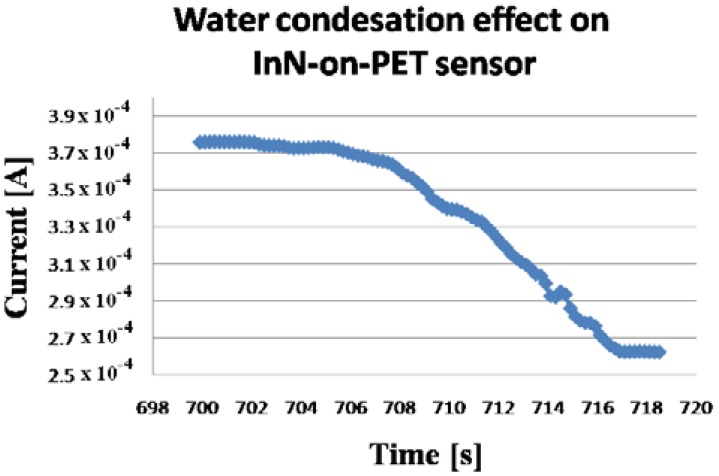
Current decrease during water condensation for an InN-on-PET condensation sensor. This corresponds to region a in Figure 10.

In a few seconds, condensation occurred on the sensors surface, forming visible water droplets. After the water cup was removed, the water droplets from the sensor surface evaporated. The procedure was repeated few times. The evolution of the measured current through the sensors is displayed in Figures 8, 9, 10 and 11. As presented in Figure 8, for the InN-on-glass device, the sensor signal is highly reproducible and follows closely the water condensation and the subsequent evaporation of the water droplets at the sensor surface. The measurements were performed in a laboratory room, with no sunlight and a constant level of artificial light only throughout the experiment. Although not specific tests were performed, no obvious influence of the illumination level was observed.

**Figure 12.**
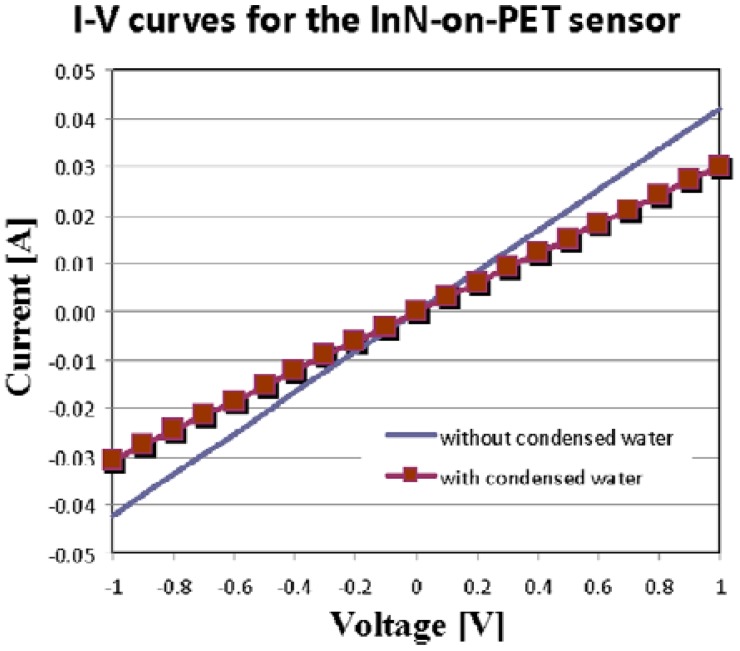
I–V characteristics of the InN-on-PET condensation sensor measured in regions b (with no condensed water) and c (with condensed water) of Figure 10 curve.

The sensor response time at water condensation is extremely fast, less than 2 s, as depicted in Figure 9. For the InN-on-PET sensor, a small drift was detected (Figure 10), and the sensor response time was close to 9 s. This difference might be due to the fact the PET layer is significantly more porous than the glass substrate. Therefore, the InN film grown on PET could have higher porosity which might affect the sensing properties. However, further investigations are needed in order to clarify this difference. In order to further investigate the sensor, the I–V characteristics displayed in Figure 12 were recorded. These measurements were performed when the current values were in regions b and c of the curve in Figure 10. Both curves are linear, indicating that the contacts are ohmic, with no rectifying behavior, and that the sensor behaves like a resistor with the resistance increasing in the presence of condensed water droplets on the its surface.

**Figure 13.**
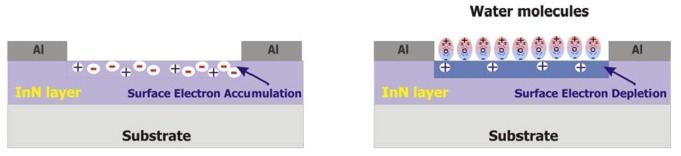
The sensing mechanism of the InN-based water condensation sensors.

Despite the significance thickness of the InN layer (1.8 μm), the sensor current exhibits a strong decrease (20%–30%) when water condenses on the exposed InN film. This suggests that the current flows mainly through the top of the InN layer and is due to the electron accumulation region near film surface, which is known to appear in InN [8–10]. Therefore, the sensing mechanism of the InN condensation sensor presented in this paper might be similar to that proposed by Lu *et al.* [8] for the anion detection with an InN-based Ion-Sensitive Field-Effect Transistor (ISFET). As schematically depicted in Figure 13, when water condensation droplets form on the open gate region of the InN sensor, they turn the electron accumulation region into a depletion one. This happens because water is a polar molecule, in which the oxygen atom induces the negative charge and the hydrogen atoms induce the positive one. Therefore, positively charged donors in the InN surface accumulation region favors the adsorption of those water molecules which have the oxygen atom oriented towards the InN surface. In this way, the electrons are repelled from the InN accumulation region turning it into a depletion one. This strongly lowers the current through the sensor, in accordance with the experimental results presented above. However, for a complete understanding of the sensing mechanism, further investigations of the InN layer surface composition will be carried out in the future. Also, other factors, such as crystalline properties, film morphology and oxidation processes may have an effect on the sensing properties and need to be investigated.

## 4. Conclusions

In conclusion, we have experimentally demonstrated and theoretically justified water condensation detection with InN deposited by sputtering on either glass or flexible plastic substrates. The measured results confirm the significant potential of InN films for sensing applications and open the way for obtaining low-cost, fast water condensation sensors, on various large area substrates, suitable for mass-production and large volume fabrication.
